# Case Series: Obstacles in the Treatment of Patients with Schizophrenia Lacking Family Support

**DOI:** 10.1192/j.eurpsy.2026.12189

**Published:** 2026-07-31

**Authors:** D. Bosnjak Kuharic, A. Jambrosic Sakoman, D. Tkalec, M. Lasic

**Affiliations:** 1 Deparment for psychotic disorders- female, University Psychiatric Hospital Vrapče, Zagreb; 2 Department for psychiatry, Varaždin General Hospital, Varaždin, Croatia

## Abstract

**Introduction:**

Family interventions play an important role in the treatment of schizophrenia, as their efficacy has been demonstrated in numerous studies and is therefore incorporated into many clinical guidelines (Caqueo-Urizar et al., *Neuropsychiatr Dis Treat* 2015;11:145–151). According to a recent systematic review and network meta-analysis (Rodolico et al., *Lancet Psychiatry* 2022;9(3):211–221), different types of family interventions have been shown to be effective in preventing relapse. Moreover, even simpler approaches, such as family psychoeducation, may be beneficial and, in some cases, superior to more complex interventions involving advanced skills or training.

**Objectives:**

Our objective was to highlight possible complications in the treatment of patients with schizophrenia who lack adequate family support.

**Methods:**

We describe a series of case reports of four female patients diagnosed with schizophrenia, including detailed medical histories, data on various treatments provided, and descriptions of family dynamics and involvement.

**Results:**

In all presented cases, the absence of appropriate family support and involvement contributed to communication difficulties, poor adherence, frequent relapses with severe and prolonged symptoms, and repeated hospitalizations. In several instances, patients “escaped” to the hospital, unable to function in their home environments.

**Conclusions:**

Family support is undeniably an important factor in achieving both symptomatic and functional remission in patients with severe mental illness. However, despite various interventions being available, limitations remain, highlighting the need for more intensive, family-inclusive treatment approaches. Contributing factors include a shortage of professional personnel, insufficient communication between systems (e.g., health care and social care) and across levels of health care (e.g., primary vs. specialist care), as well as limited availability of community services.

**Disclosure of Interest:**

None Declared

